# Frequency of Fish Intake and Diabetes among Adult Indians

**DOI:** 10.1080/07315724.2013.867420

**Published:** 2014-05-28

**Authors:** Sutapa Agrawal, Christopher Millett, S. V. Subramanian, Shah Ebrahim

**Affiliations:** ^a^ South Asia Network for Chronic Disease, Public Health Foundation of India, New Delhi, INDIA; ^b^ Department of Primary Care and Public Health, School of Public Health, Imperial College, London, UK; ^c^ Department of Society, Human Development and Health, Harvard School of Public Health, Harvard University, Boston; ^d^ Department of Non-communicable Disease Epidemiology, London School of Hygiene and Tropical Medicine, London, UK

**Keywords:** frequency of fish intake, self-reported diabetes, men, women, NFHS-3, India

## Abstract

**Objectives:** Recent studies have shown that the choice of foods plays a role in diabetes prevention. However, little empirical evidence on this association exists in developing countries. We aimed to examine the association between frequency of fish intake and self-reported diabetes status among adult men and women in India.

**Methods:** Analysis of cross-sectional data from participants in India's third National Family Health Survey conducted during 2005–2006 was performed. Associations between fish intake, determined by frequency of consumption (daily, weekly, occasionally, and never), and self-reported diabetes were estimated using multivariable-adjusted models in 99,574 women, 56,742 men, and 39,257 couples aged 20–49 years after adjusting for frequency of consumption of other food items, body mass index (BMI) status, tobacco smoking, alcohol drinking, watching television, age, education, living standard of the household, and place of residence.

**Results:** After adjustment for other dietary, lifestyle, and socioeconomic and demographic characteristics, odds of diabetes were 2 times higher (odds ratio [OR]: 2.02; 95% confidence interval [CI], 1.59–2.57; *p* < 0.0001) among those who reported consuming fish daily compared to those who never consumed fish. Weekly fish intake was also associated with a higher odds of having diabetes (OR: 1.55; 95% CI, 1.25–1.93; *p* < 0.0001). The adjusted effect of daily fish intake on diabetes was greater among men (OR: 2.46; 95% CI, 1.66–3.65) than among women (OR: 1.72; 95% CI, 1.26–2.33). In cross-spousal sensitivity analysis, the odds of a husband having diabetes was also associated with wife's daily/weekly consumption of fish (OR: 1.36; 95% CI, 0.92–2.01) and the odds of a wife having diabetes was also associated with husband's daily/weekly consumption of fish (OR: 1.21; 95% CI, 0.87–1.68).

**Conclusions:** In a large nationally representative sample of adult men and women in India, daily or weekly fish intake was positively associated with the presence of diabetes. However, this is an observational finding and uncontrolled confounding cannot be excluded as an explanation for the association. More epidemiological research with better measures of food intake and clinical measures of diabetes is needed in a developing country setting to validate the findings.

## INTRODUCTION

A prudent diet is a key component of a healthy lifestyle for preventing type 2 diabetes [1,2]. Though fish, particularly oily fish, is generally considered to be an important part of a healthy diet, concerns have been raised that fish consumption is associated with a higher risk of developing diabetes [3,4]. However, the emerging scientific evidence for this association is not consistent and most previous studies have been conducted in developed countries. For example, fish and seafood consumption have been associated with low prevalence of chronic diseases (including diabetes) in Greenland Inuit populations consuming a predominantly marine diet [5]. Cohort studies have shown protective effects of fish intake against the development of diabetes in the elderly [6,7], and an ecological study similarly reported a protective effect in populations with a high prevalence of obesity [8]. Other prospective studies suggested that white and oily fish consumption may be beneficial for reducing risk of diabetes [9] but shellfish intake seems to be associated with an increased risk [9]. Findings from cross-sectional studies have reported inverse [10–12], no [13], or positive [4,14] associations between habitual fish intake and diabetes.

Despite a growing prevalence of type 2 diabetes in Indian populations, in both rural [15,16] and urban areas [17–20], there is little empirical evidence on the specific food items that may be contributing to this, including the potential role of fish consumption. The independent association between frequency of fish intake and diabetes prevalence is not well documented, particularly in developing countries. India's third National Family Health Survey (NFHS-3, 2005–2006) collected data from 109,041 households on a wide range of dietary, societal, lifestyle, and environmental determinants of morbidity and chronic ailments including diabetes [21] and provides a unique opportunity to study the association between frequency of fish consumption and the prevalence of diabetes in a large, nationally representative sample.

**Figure:**
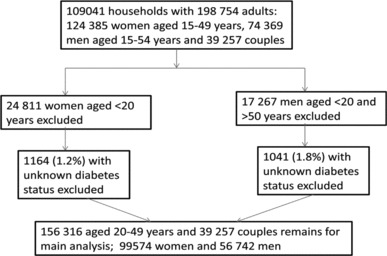
Flow diagram showing exclusions and final sample sizes for the third National Family Health Survey (NFHS-3) analysis.

## MATERIALS AND METHODS

### Data

Data from the NFHS-3 (2005–2006) were used for this study. Details of survey objective, survey method including sampling frame, and questionnaire used are provided in the survey report [21]. Briefly, this survey (also available at http://www.iipsindia.org) was designed on the lines of the Demographic and Health Surveys (DHS; available at http://www.measuredhs.com) that have been conducted in over 90 developing countries since the 1980s. NFHS-3 collected demographic, socioeconomic, and health information from a nationally representative probability sample of 124,385 women aged 15–49 years and 74,369 men aged 15–54 years residing in 109,041 households. The sample is a multistage cluster sample with an overall response rate of 98% (95% for women and 87% for men) [21]. All states of India are represented in the sample (except the small Union Territories), covering more than 99% of country's population. The analysis presented here is restricted to the 99,574 women and 56,742 men aged 20–49 years living in the sample households. We excluded age below 20 years to avoid any cases of childhood diabetes for which the etiology and risk factors might be different; age above 50 years is also excluded for comparison purpose because information for women age above 50 years was not collected in the survey. Fig. 1 shows the exclusions and final sample sizes for the analysis.

**Outcome Evaluation.** The survey asked participants the question, “Do you currently have diabetes?” with response options of y*es*, *no*, and *don't know*. Data on physician-reported diagnosis of diabetes and fasting blood glucose were available in the NFHS-3 to verify a self-reported diagnosis.

**Dietary Predictor Variables and Covariates.** The survey collected information on demographic and socioeconomic factors, anthropometric measurements, and dietary intake through personal interview. Consumption of selected foods was assessed by asking, “How often do you yourself consume the following items: daily, weekly, occasionally or never?” related to fish consumption, milk or curd, pulses or beans, green leafy vegetables, other vegetables and fruits, eggs, chicken, or meat. Frequency of watching television (almost every day, at least once weekly, less than once weekly, not at all) was used as a measure of sedentary behavior. The survey also collected information on the use of tobacco directly by asking respondents to report on their own tobacco use. All eligible men and women who were interviewed were asked 4 specific questions on current use of tobacco (smoke and nonsmoker variants): “Do you currently smoke cigarettes or *bidis*?”; “In the last 24 hours, how many cigarettes or *bidis* have you smoked?”; “Do you currently smoke or use tobacco in another form?”; and “In what other form do you currently smoke or use tobacco?” The information from these 4 questions was used to ascertain exposure to tobacco smoke: yes, active smoker (person currently smokes) and nonsmoker (the person has never smoked). However, past smoking was not ascertained from the respondents. Use of alcohol was quantified as drinks taken almost every day, about once weekly, less than once weekly, and never.

Respondents were weighed using a solar-powered digital scale (SECA 874 digital scale; Seca GmbH & Co., Hamburg, Germany) with an accuracy of ±100 g [22]. Their height was measured using an adjustable wooden measuring board (Shorr height board), specifically designed to provide accurate measurements (to the nearest 0.1 cm) [22]. Indian adult population standard [23–25] categories of body mass index (BMI, kg/m^2^) were used: ≤18.5 kg/m^2^ (underweight); 18.5–22.9 kg/m^2^ (normal), 23.0–24.9 kg/m^2^ (overweight), and ≥25.0 kg/m^2^ (obese). Other covariates in our analysis included age (20–29, 30–39, 40–49 years); education (illiterate, literate but less than middle school complete, middle school complete but less than high school complete, high school complete or higher); religion (Hindu, Muslim, Christian, Sikh, Others); caste/tribe status (scheduled caste, scheduled tribe, other backward class, others); wealth status (based on 33 assets and housing characteristics graded lowest, second, middle, fourth, highest); and place of residence (urban, rural). For a detailed definition of variables see Table 1.

**Table 1:** Sample Distribution and Prevalence of Diabetes (%) among Men (*n* = 56,742) and Women (*n* = 99,574) Age 20–49 Years According to Frequency of Fish Intake and Other Selected Risk Factors and Background Characteristics, India, 2005–2006

	Men			Women			Total		
Characteristics	Subjects, *N* (%)	Diabetes, *N* (%)	χ^2^*p* Value	Subjects, *N* (%)	Diabetes, *N* (%)	χ^2^*p* value	Subjects, *N* (%)	Diabetes, *N* (%)	χ^2^*p* Value
Frequency of fish intake			<0.0001			<0.0001			<0.0001
Daily	3706 (6.5)	90 (2.4)		6505 (6.5)	149 (2.3)		10211 (6.5)	240 (2.4)	
Weekly	14,414 (25.4)	238 (1.7)		22,070 (22.2)	304 (1.4)		36484 (23.3)	542 (1.5)	
Occasionally	21,818 (38.5)	225 (1.0)		34,242 (34.4)	264 (0.8)		56060 (35.9)	489 (0.9)	
Never	16,782 (29.6)	167 (1.0)		36,724 (36.9)	331 (0.9)		53506 (34.2)	498 (0.9)	
Frequency of intake of other food items									
Milk or curd			<0.0001			<0.0001			<0.0001
Daily	26,307 (46.4)	391 (1.5)		40,366 (40.5)	492 (1.2)		66673 (42.7)	883 (1.3)	
Weekly	11,554 (20.4)	117 (1.0)		15,071 (15.1)	138 (0.9)		26626 (17.0)	255 (1.0)	
Occasionally	14,757 (26.0)	138 (0.9)		32,918 (33.1)	302 (0.9)		47675 (30.5)	440 (0.9)	
Never	4114 (7.3)	74 (1.8)		11,202 (11.3)	117 (1.0)		15317 (9.8)	191 (1.2)	
Pulses and beans			<0.0001			<0.0001			<0.0001
Daily	29,863 (52.6)	437 (1.5)		52,440 (52.7)	538 (1.0)		82303 (52.7)	975 (1.2)	
Weekly	21,705 (38.3)	219 (1.0)		36,597 (36.8)	360 (1.0)		58302 (37.3)	579 (1.0)	
Occasionally	4660 (8.2)	51 (1.1)		9663 (9.7)	131 (1.4)		14323 (9.2)	182 (1.3)	
Never	505 (0.9)	13 (2.6)		852 (0.9)	20 (2.3)		1357 (0.9)	33 (2.4)	
Green leafy vegetables			0.149			0.090			0.368
Daily	33,982 (59.9)	453 (1.3)		64,095 (64.4)	674 (1.1)		98076 (62.7)	1127 (1.1)	
Weekly	19,270 (34.0)	231 (1.2)		28,606 (28.7)	286 (1.0)		47876 (30.6)	517 (1.1)	
Never/occasionally	3480 (6.1)	35 (1.0)		6840 (6.9)	89 (1.3)		10321 (6.6)	125 (1.2)	
Fruits			<0.0001			<0.0001			<0.0001
Daily	7320 (12.9)	125 (1.7)		12,789 (12.9)	206 (1.6)		20109 (12.9)	331 (1.6)	
Weekly	19,368 (34.1)	255 (1.3)		26,731 (26.9)	276 (1.0)		46099 (29.5)	531 (1.2)	
Occasionally	28,484 (50.2)	296 (1.0)		56,336 (56.6)	503 (0.9)		84820 (54.3)	800 (0.9)	
Never	1546 (2.7)	44 (2.8)		3631 (3.6)	63 (1.7)		5177 (3.3)	107 (2.1)	
Eggs			<0.0001			<0.0001			<0.0001
Daily	2931 (5.2)	56 (1.9)		3475 (3.5)	60 (1.7)		6405 (4.1)	115 (1.8)	
Weekly	20,682 (36.5)	317 (1.5)		28,778 (28.9)	363 (1.3)		49460 (31.6)	680 (1.4)	
Occasionally	19,786 (34.9)	201 (1.0)		32,635 (32.8)	287 (0.9)		52421 (33.5)	488 (0.9)	
Never	13,330 (23.5)	146 (1.1)		34,647 (34.8)	340 (1.0)		47977 (30.7)	486 (1.0)	
Chicken or meat			<0.0001			<0.0001			<0.0001
Daily	706 (1.2)	6 (0.9)		839 (0.8)	14 (1.7)		1545 (1.0)	20 (1.3)	
Weekly	15,609 (27.5)	269 (1.7)		21,938 (22.0)	292 (1.3)		37548 (24.0)	561 (1.5)	
Occasionally	26,135 (46.1)	291 (1.1)		42,222 (42.0)	423 (1.0)		68357 (43.7)	714 (1.0)	
Never	14,272 (25.2)	155 (1.1)		34,537 (34.7)	320 (0.9)		48809 (31.2)	475 (1.0)	
Body mass index and lifestyle factors									
Body mass index (kg/m^2^)^a^			<0.0001			<0.0001			<0.0001
≤18.5 (Underweight)	11,896 (22.2)	96 (0.8)		24,991 (26.2)	119 (0.5)		36887 (24.8)	215 (0.6)	
18.5–22.9 (Normal)	30,076 (56.2)	288 (1.0)		46,892 (49.1)	319 (0.7)		76968 (51.7)	606 (0.8)	
23.0–24.9 (Overweight)	5635 (10.5)	128 (2.3)		9454 (9.9)	153 (1.6)		15089 (10.1)	281 (1.9)	
≥25.0 (Obese)	5881 (11.0)	178 (3.0)		14,169 (14.8)	437 (3.1)		20050 (13.5)	615 (3.1)	
Current Tobacco smoking^b^			0.498			0.514			0.038
No	35,422 (62.4)	450 (1.3)		97,738 (98.2)	1030 (1.1)		133160 (85.2)	1480 (1.1)	
Yes	21,321 (37.6)	270 (1.3)		1835 (1.8)	19 (1.0)		223156 (14.8)	289 (1.2)	
Alcohol consumption			0.362			0.020			0.107
Never	35,965 (63.4)	436 (1.2)		97,101 (97.5)	1037 (1.1)		133067 (85.1)	1473 (1.1)	
Occasionally	13,054 (23.0)	180 (1.4)		1067 (1.1)	7 (0.7)		14121 (9.0)	187 (1.3)	
Once a week	5676 (10.0)	74 (1.3)		1010 (1.0)	3 (0.3)		6686 (4.3)	77 (1.2)	
Almost every day	2048 (3.6)	31 (1.5)		396 (0.4)	1 (0.3)		2443 (1.6)	32 (1.3)	
Frequency of watching TV			<0.0001			<0.0001			<0.0001
Not at all	10,517 (18.5)	112 (1.1)		35,399 (35.6)	255 (0.7)		45916 (29.4)	366 (0.8)	
Less than once a week	11,420 (20.1)	95 (0.8)		10,438 (10.5)	96 (0.9)		21859 (14.0)	191 (0.9)	
At least once a week	9081 (16.0)	114 (1.3)		10,952 (11.0)	100 (0.9)		20033 (12.8)	213 (1.1)	
Almost every day	25,717 (45.3)	400 (1.6)		42,763 (43.0)	598 (1.4)		68480 (43.8)	999 (1.5)	
*(Continued on next page)*

**Table NaN:** Sample Distribution and Prevalence of Diabetes (%) among Men (*n* = 56,742) and Women (*n* = 99,574) Age 20–49 Years According to Frequency of Fish Intake and Other Selected Risk Factors and Background Characteristics, India, 2005–2006 *(Continued)*

	Men			Women			Total		
Characteristics	Subjects, *N* (%)	Diabetes, *N* (%)	χ^2^*p* Value	Subjects, *N* (%)	Diabetes, *N* (%)	χ^2^*p* value	Subjects, *N* (%)	Diabetes, *N* (%)	χ^2^*p* Value
Background factors									
Age			<0.0001			<0.0001			<0.0001
20–29	22,842 (40.3)	91 (0.4)		43,196 (43.4)	113 (0.3)		66038 (42.2)	204 (0.3)	
30–39	19,045 (33.6)	179 (0.9)		33,522 (33.7)	342 (1.0)		52567 (33.6)	520 (1.0)	
40–49	14,855 (26.2)	450 (3.0)		22,856 (23.0)	594 (2.6)		37711 (24.1)	1045 (2.8)	
Education^c^			<0.0001			<0.0001			<0.0001
Illiterate	11,607 (20.5)	144 (1.2)		45,113 (45.3)	338 (0.7)		56720 (36.3)	482 (0.9)	
Literate, less than middle school	10,030 (17.7)	111 (1.1)		14,463 (14.5)	192 (1.3)		24493 (15.7)	303 (1.2)	
Middle school completed	26,783 (47.2)	320 (1.2)		31,665 (31.8)	435 (1.4)		58448 (37.4)	754 (1.3)	
High school complete and above	8311 (14.7)	146 (1.8)		83,284 (8.4)	83 (1.0)		16639 (10.6)	229 (1.4)	
Religion			0.099			<0.0001			<0.0001
Hindu	46,727 (82.3)	575 (1.2)		80,648 (81.0)	792 (1.0)		127375 (81.5)	1367 (1.1)	
Muslim	6841 (12.1)	103 (1.5)		12,940 (13.0)	164 (1.3)		19781 (12.7)	267 (1.4)	
Christian	1290 (2.3)	19 (1.5)		2526 (2.5)	56 (2.2)		3816 (2.4)	75 (2.0)	
Sikhs	1009 (1.8)	17 (1.7)		1836 (1.8)	21 (1.1)		2845 (1.8)	38 (1.3)	
Other^d^	876 (1.5)	6 (0.7)		1624 (1.6)	16 (1.0)		2500 (1.6)	22 (0.9)	
Caste/tribe^e^			<0.0001			<0.0001			<0.0001
Scheduled caste	10,670 (18.8)	131 (1.2)		18,260 (18.3)	173 (0.9)		28931 (18.5)	304 (1.1)	
Scheduled tribes	4732 (8.3)	24 (0.5)		8002 (8.0)	30 (0.4)		12734 (8.1)	54 (0.4)	
Other backward class	22,116 (39.0)	256 (1.2)		38,860 (39.0)	368 (0.9)		60977 (39.0)	624 (1.0)	
Others	17,414 (30.7)	270 (1.6)		31,440 (31.6)	437 (1.4)		48854 (31.3)	706 (1.4)	
Missing caste	1810 (3.2)	40 (2.2)		3011 (3.0)	41 (1.4)		4821 (3.1)	81 (1.7)	
Wealth index^f^			<0.0001			<0.0001			<0.0001
Lowest	9103 (16.0)	71 (0.8)		17,286 (17.4)	71 (0.4)		26389 (16.9)	142 (0.5)	
Second	10,205 (18.0)	100 (1.0)		18,546 (18.6)	141 (0.8)		28751 (18.4)	241 (0.8)	
Middle	11,533 (20.3)	80 (0.7)		19,698 (19.8)	152 (0.8)		31232 (20.0)	232 (0.7)	
Fourth	12,634 (22.3)	154 (1.2)		20,925 (21.0)	275 (1.3)		33560 (21.5)	428 (1.3)	
Highest	13,266 (23.4)	316 (2.4)		23,119 (23.2)	411 (1.8)		36385 (23.3)	726 (2.0)	
Place of residence			<0.0001			<0.0001			<0.0001
Urban	20,779 (36.6)	347 (1.7)		33,355 (33.5)	551 (1.7)		54134 (34.6)	898 (1.7)	
Rural	35,963 (63.4)	373 (1.0)		66,219 (66.5)	498 (0.8)		102183 (65.4)	871 (0.9)	
Total percentage		1.3			1.1			1.1	
Number^g^	56,742	720		99,574	1050		156316	1769 (1.1)	

^a^Women who were pregnant at the time of the survey or women who had given birth during the 2 months preceding the survey were excluded from these measurements.^b^The survey also collected information on use of tobacco directly by asking respondents to report on their own tobacco use. Four specific questions (“Do you currently smoke cigarettes or *bidis*?”; “In the last 24 hours how many cigarettes or *bidis* did you smoke?”; “Do you currently smoke or use tobacco in other form?”; and “In what other form do you currently smoke or use tobacco?”) on current use of tobacco (smoked and nonsmoked variants) were asked of all eligible men and women who were interviewed. The information from these 4 questions was used to ascertain exposure to tobacco smoke—yes, active smoking (person currently smokes) and no smoking (the person has never smoked).^c^Education: illiterate (0 years of education), literate but less than middle school complete (1–5 years of education), middle school complete (6–8 years of education), high school complete or more (9+ years of education).^d^Others include Sikh, Buddhist, Christian, Jain, Jewish, Zoroastrian.^e^Scheduled castes and scheduled tribes are identified by the government of India as socially and economically backward and needing protection from social injustice and exploitation. Other backward class is a diverse collection of intermediate castes that were considered low in the traditional caste hierarchy but are clearly above scheduled castes. Others is thus a default residual group that enjoys higher status in the caste hierarchy.^f^The wealth index has been developed and tested in a large number of countries in relation to inequalities in household income, use of health services, and health outcomes. It is an indicator of the level of wealth that is consistent with expenditure and income measures. The economic index was constructed using household asset data and housing characteristics such as household electrification; type of windows; drinking water source; type of toilet facility; type of flooring; material of exterior walls; type of roofing; cooking fuel; house ownership; number of household members per sleeping room; ownership of a bank or post office account; and ownership of a mattress, pressure cooker, chair, cot/bed, table, electric fan, radio/transistor, black-and-white television, color television, sewing machine, mobile telephone, any other telephone, computer, refrigerator, watch or clock, bicycle, motorcycle or scooter, animal-drawn cart, car, water pump, thresher, and tractor.^g^Number of men and women varies slightly for individual variables depending on the number of missing values.

### Statistical Analyses

Descriptive statistics were calculated with use of standard methods (such as frequencies and percentages) in men and women separately. Categorical variables are presented as absolute and relative frequencies. Associations between categorical variables were tested by the calculation of the chi-square test. Multivariable logistic regression models were used to estimate the odds ratios of daily and weekly fish intake on diabetes prevalence after controlling for potential confounders and also examining the independent effects of other potential risk factors. To adjust for potentially confounding factors, we used multivariate logistic regression models, as follows:

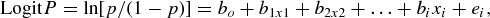
where *b*_1_, *b*_2_, …, *b_i_* represent the coefficient of each of the independent variables included in the model, and *e_i_* is an error term. Ln[*p*/(1 − *p*)] represents the natural logarithms of the odds of the outcome variable or dependent variable, which in this case is diabetes. The odds ratios are thus the measures of odds of diabetes prevalence (response variable) as indicated by the main predictor variable (independent variable) such as frequency of fish intake (0 = never, 1 = daily, 2 = weekly, 3 = occasionally) and other dietary (all coding same as fish intake) and socioeconomic and demographic confounders and effect modifiers such as BMI, smoking, alcohol consumption, and TV watching. This model has been used to elicit the net effects of each of the explanatory variable while accounting for the other predictor variables under analysis on the likelihood of suffering from diabetes. The response variable (self-reported diabetes) has been categorized into two mutually exclusive and exhaustive categories: respondents reporting suffering from diabetes (coded as 1) or respondents reporting not suffering from diabetes (coded as 0). With regard to the direction of logit coefficients, odds greater than 1 indicate an increased probability that women will have a high chance of suffering from diabetes, whereas odds greater than 1 one indicate a decreased probability. The logistic regression equation estimates the effect of one unit change in the independent variable (when *x* is discrete) on the logarithm of odds (log-odds) that the dependent variable takes when controlled for the effects of other independent variables [26–28]. The parameters in the logistic models were estimated using the maximum likelihood method.

The following models were constructed to account for potential confounders and mediators. Model 1 presents unadjusted results; model 2 presents results adjusted for the frequency of consumption of other food items (milk or curd, pulses or beans, green leafy vegetables, other vegetables and fruits, eggs, chicken, or meat); model 3 included additional adjustment for current tobacco smoking, alcohol consumption, TV watching, and BMI because these factors may mediate the association between fish intake and diabetes; model 4 is adjusted for all risk factors, mediators, and confounders to demonstrate any independent effect of fish consumption. We also conducted a cross-spousal sensitivity analysis to examine this association in couples (*n* = 39,257). We examined a possible interaction between frequency of fish intake and sex by using a log-likelihood ratio test in model 4 and found the interaction to be significant (*p* < 0.0001). The log-likelihood ratio test (lrt) is performed by estimating two models and comparing the fit of one model to the fit of the other. Removing predictor variables from a model will almost always make the model fit less well (i.e., a model will have a lower log likelihood), but it is necessary to test whether the observed difference in model fit is statistically significant. The lrt does this by comparing the log likelihoods of the two models; if this difference is statistically significant, then the less restrictive model (the one with more variables) is said to fit the data significantly better than the more restrictive model. If one has the log likelihoods from the models, the lrt is fairly easy to calculate. The formula for the lrt statistic is

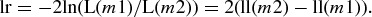



Therefore, we presented the adjusted results for both the total sample and adjusted analysis stratified by sex. All reported *p* values were based on 2-sided tests. Because certain states and certain groups of respondents were oversampled in the survey, sample weights were used to restore the representativeness of the sample [21].

Results are presented in the form of odds ratios (ORs) with 95% confidence intervals (CIs). The estimation of confidence intervals takes into account the design effects due to clustering at the level of the primary sampling unit. Before carrying out the multivariate models, we tested for the possibility of multicolinearity between the variables. In the correlation matrix, all pairwise Pearson correlation coefficients are <0.5, suggesting that multicolinearity is not a problem. All analyses including the logistic regression models were conducted using the Statistical Package for Social Sciences software version 19.0 (IBM SPSS Statistics, Chicago, IL).

### Human Subject Informed Consent

The analysis is based on secondary analysis of existing survey data with all identifying information removed. The NFHS-3 survey was approved by the ethical review boards of the implementing agencies and the Indian government. Participation in the survey was totally voluntary. The survey obtained written informed consent from each respondent (in this case, men and women included in the analysis) before asking questions and separately before obtaining height and weight.

## RESULTS

A very low percentage (6.5%) of respondents reported daily fish intake; almost one-fourth (23.3%) reported weekly fish intake and more than one-third (34.2%) reported never consuming fish (see Appendix, Table A1). Daily fish intake was more common in respondents in the youngest (20–29) age group than in the oldest (40–49) age group (40.1% vs 25.1%; *p* < 0.0001) and those with higher socioeconomic status, living in rural areas compared to urban areas (61.4% vs 39.9%; *p* < 0.0001), those in households with the highest wealth quintile compared to lowest wealth quintile (31.9% vs 8.2%; *p* < 0.0001), among respondents belonging to other caste/tribe status category compared to low caste (44.0% vs 3.4%; *p* < 0.0001), and among those who completed middle school education compared to those with higher education (50.5% vs 15.4; *p* < 0.0001; see Appendix, Table A2).

The overall prevalence of diabetes was higher in men than women (1.3% vs 1.1%; *p* < 0.0001; Table 1). Men and women who consumed fish daily (2.4% and 2.3%, respectively) or weekly (1.7% and 1.4%, respectively) were more (*p* < 0.0001) likely to have diabetes than those who never consumed fish (1.0% and 0.9%, respectively). Diabetes was more common among both men and women who consumed milk or curd, eggs, chicken, or meat daily or weekly, never consumed pulses and beans or fruits, were either overweight or obese, watched television almost every day, were in the oldest age group, lived in urban areas, and lived in wealthier households (all *p* < 0.0001). Higher associations (*p* < 0.0001) between age and diabetes prevalence were observed. Diabetes prevalence increased with increasing household wealth and was almost double in urban women and men compared to their rural counterparts.

Table 2 shows associations between daily and weekly fish consumption and diabetes in unadjusted, partially adjusted, and fully adjusted models. In the unadjusted analysis, odds of having diabetes were 2.6 times higher (OR = 2.56; 95% CI, 2.19–2.99) among those who consumed fish daily and 1.6 times higher (OR = 1.61; 95% CI, 1.42–1.81) among those who consumed fish weekly than those who never consumed fish. Controlling for consumption of other food items (in model 2) reduces the effect of daily fish intake on diabetes prevalence (OR = 2.03; 95% CI, 1.61–2.57). The effect of daily fish intake remains virtually unchanged (OR = 2.13; 95% CI, 1.80–2.52) when BMI and other lifestyle factors are additionally controlled in model 3. Even when the socioeconomic control variables and other risk factors are included in model 4, the effect of daily (OR = 2.02; 95% CI, 1.59–2.57) or weekly (OR = 1.55; 95% CI, 1.25–1.93) fish consumption still has a large and statistically significant (*p* < 0.0001) effect on the prevalence of diabetes.

**Table 2:** Unadjusted and Adjusted Effect (Odds Ratios with 95% CI) of Fish Consumption and Other Selected Factors on Prevalence of Diabetes, India, 2005–2006^a^

Predictors and Confounders	Model 1 OR (95% CI)	Model 2 OR (95% CI)	Model 3 OR (95% CI)	Model 4 OR (95% CI)
Frequency of fish intake				
Daily	2.56 (2.19–2.99)	2.03 (1.61–2.57)	2.13 (1.80–2.52)	2.21 (1.68–2.92)
Weekly	1.61 (1.42–1.81)	1.56 (1.26–1.93)	1.67 (1.46–1.90)	1.86 (1.31–2.65)
Occasionally	0.94 (0.83–1.06)	1.09 (0.88–1.35)	1.12 (0.98–1.28)	1.44 (0.91–2.28)
Never^b^	1.00 (Reference)	1.00 (Reference)	1.00 (Reference)	1.00 (Reference)
Frequency of intake of other food items				
Milk or curd				
Daily		1.00 (0.84–1.19)		0.95 (0.80–1.14)
Weekly		0.82 (0.67–1.00)		0.82 (0.67–1.00)
Occasionally		0.83 (0.69–0.99)		0.83 (0.69–0.99)
Never^b^		1.00 (Reference)		1.00 (Reference)
Pulses and beans				
Daily		0.53 (0.37–0.77)		0.56 (0.39–0.81)
Weekly		0.49 (0.34–0.71)		0.50 (0.35–0.72)
Occasionally		0.61 (0.42–0.89)		0.63 (0.43–0.93)
Never^b^		1.00 (Reference)		1.00 (Reference)
Green leafy vegetables				
Daily		0.94 (0.77–1.15)		0.94 (0.77–1.15)
Weekly		1.02 (0.83–1.26)		1.03 (0.84–1.27)
Never/occasionally		1.00 (Reference)		1.00 (Reference)
Fruits				
Daily		0.39 (0.30–0.50)		0.38 (0.30–0.49)
Weekly		0.36 (0.28–0.45)		0.35 (0.28–0.45)
Occasionally		0.42 (0.34–0.52)		0.43 (0.34–0.53)
Never^b^		1.00 (Reference)		1.00 (Reference)
Eggs				
Daily		1.23 (0.95–1.60)		1.14 (0.87–1.49)
Weekly		1.12 (0.92–1.35)		1.11 (0.91–1.35)
Occasionally		0.98 (0.81–1.18)		0.99 (0.82–1.20)
Never^b^		1.00 (Reference)		1.00 (Reference)
Chicken or meat				
Daily		0.72 (0.43–1.19)		0.62 (0.36–1.06)
Weekly		1.03 (0.81–1.31)		1.03 (0.81–1.31)
Occasionally		1.05 (0.84–1.31)		1.02 (0.81–1.28)
Never^b^		1.00 (Reference)		1.00 (Reference)
Body mass index and lifestyle factors				
Body mass index (kg/m^2^)				
≤18.5 (Underweight)			0.84 (0.72–0.99)	0.84 (0.72–0.99)
18.5–22.9 (Normal)^b^			1.00 (Reference)	1.00 (Reference)
23.0–24.9 (Overweight)			1.67 (1.44–1.94)	1.66 (1.43–1.92)
≥25.0 (Obese)			2.26 (1.99–2.56)	2.25 (1.98–2.56)
Current tobacco smoking				
No^b^			1.00 (Reference)	1.00 (Reference)
Yes			0.97 (0.83–1.13)	0.96 (0.82–1.12)
Alcohol consumption				
Never^b^			1.00 (Reference)	1.00 (Reference)
Occasionally			1.07 (0.89–1.28)	1.08 (0.90–1.30)
Once a week			0.92 (0.71–1.19)	0.91 (0.70–1.17)
Almost every day			0.96 (0.66–1.40)	0.96 (0.66–1.40)
Frequency of watching TV				
Not at all^b^			1.00 (Reference)	1.00 (Reference)
Less than once a week			0.89 (0.74–1.06)	0.92 (0.77–1.11)
At least once a week			0.94 (0.78–1.13)	0.98 (0.81–1.18)
Almost every day			0.90 (0.77–1.05)	0.93 (0.79–1.09)
Number of cases	157090		148564	148399

OR = odds ratio, CI = confidence interval.^a^For variable definition see Table 1.^b^Reference category; model 1 unadjusted; model 2 adjusted for consumption of other food items; model 3 adjusted for BMI and other lifestyle indicators; model 4 adjusted for models 2 and 3 additionally controlling for background factors age, gender, education, religion, caste/tribe, wealth status, and place of residence.

Associations between frequency of fish consumption and diabetes stratified by sex are presented in Table 3. The adjusted effect of daily (men: OR = 2.46; 95% CI, 1.66–3.65; women: OR = 1.72; 95% CI, 1.26–2.33) and weekly (men: OR = 1.77; 95% CI, 1.24–2.53; women: OR = 1.41; 95% CI, 1.07–1.87) fish consumption on the prevalence of diabetes was statistically significant in both men and women although the odds ratio was greater in men.

**Table 3:** Unadjusted and Adjusted Effect (Odds Ratios with 95% CI) of Frequency of Fish Intake and Other Selected Factors on the Prevalence of Diabetes among Men (*n* = 56,742) and Women (*n =* 99,574), India, 2005–2006

Predictors and Confounders	Model 1 OR (95% CI)	Model 2 OR (95% CI)	Model 3 OR (95% CI)	Model 4 OR (95% CI)
Frequency of fish intake in men				
Daily	2.48 (1.92–3.22)	2.38 (1.62–3.49)	2.35 (1.77–3.12)	2.46 (1.66–3.65)
Weekly	1.67 (1.37–2.03)	1.75 (1.24–2.47)	1.93 (1.55–2.40)	1.77 (1.24–2.53)
Occasionally	1.04 (0.85–1.27)	1.33 (0.95–1.86)	1.27 (1.02–1.59)	1.37 (0.97–1.94)
Never^a^	1.00 (Reference)	1.00 (Reference)	1.00 (Reference)	1.00 (Reference)
Frequency of fish intake in women				
Daily	2.58 (2.13–3.14)	1.78 (1.32–2.40)	1.93 (1.56–2.39)	1.72 (1.26–2.33)
Weekly	1.54 (1.32–1.80)	1.42 (1.08–1.88)	1.50 (1.26–1.77)	1.41 (1.07–1.87)
Occasionally	0.86 (0.73–1.01)	0.94 (0.71–1.24)	1.03 (0.86–1.22)	0.94 (0.71–1.25)
Never^a^	1.00 (Reference)	1.00 (Reference)	1.00 (Reference)	1.00 (Reference)

OR = odds ratio, CI = confidence interval.^a^Reference category; model 1 unadjusted; model 2 adjusted for consumption of other food items; model 3 adjusted for BMI and other lifestyle indicators; model 4 adjusted for models 2 and 3 additionally controlling for background factors age, gender, education, religion, caste/tribe, wealth status, and place of residence.

Bivariate association between wives’ and husbands’ fish consumption pattern in India is presented in Table A3 in the Appendix and findings from the cross-spousal multivariable regression analysis on 39,257 couples are presented in and Table 4. The adjusted odds of a husband having diabetes was 1.15 (95% CI, 0.76–1.74) when he consumed fish daily or weekly; the odds increased to 1.4 times more (95% CI, 0.92–2.01) when his wife consumed fish but he did not in comparison to those couples who never consumed fish. The adjusted odds of a wife having diabetes was 1.4 times higher (95% CI, 0.98–2.02) when she consumed fish daily/weekly and was 1.2 times more (95% CI, 0.87–1.68) when her husband consumed fish but she did not.

**Table 4:** Prevalence of Diabetes and Unadjusted and Adjusted Odds Ratios (with 95% CI) Showing the Association between Cross-Spousal Fish Consumption and Couples’ (*n* = 39,257) Diabetes Status, India, 2005–2006

	Diabetes Prevalence,	Unadjusted OR	Adjusted OR
Daily/Weekly Fish Consumption	*N* (%)	(95% CI)	(95% CI)
Wife's frequency of fish intake on husband's diabetes status	67 (1.8)	1.23 (0.89–1.98)	1.36 (0.92–2.01)
Husband's frequency of fish intake on wife's diabetes status	56 (1.2)	1.30 (0.89–1.96)	1.21 (0.87–1.68)
Wife's frequency of fish intake on wife's diabetes status	55 (1.4)	1.53 (0.95–1.98)	1.41 (0.98–2.02)
Husband's frequency of fish intake on husband's diabetes status	89 (2.0)	1.37 (0.85–1.85)	1.15 (0.76–1.74)
Wife's + husband's frequency of fish intake and husband's diabetes status	221 (2.9)	2.02 (1.54–2.24)	1.55 (1.12–2.14)
Wife's + husband's frequency of fish intake and wife's diabetes status	155 (1.9)	2.09 (1.21–2.23)	1.55 (1.17–2.05)
Wife's + husband both never consuming fish^a^	—	1.00 (Reference)	1.00 (Reference)

OR = odds ratio, CI = confidence intervals.^a^Reference category; adjusted odds ratios potentially controlled for consumption of other food items, body mass index, and other lifestyle indicators and background factors including age, gender, education, religion, caste/tribe, wealth status, and place of residence.

## DISCUSSION

The results of this nationally representative cross-sectional study do not support the hypothesis that fish intake is protective against diabetes in adult Indian populations. Instead, we observed a significantly higher likelihood of diabetes among respondents consuming fish either daily or weekly when compared to those who do not eat fish. The association is robust after controlling for other risk factors such as consumption of other food items, BMI, tobacco smoking, alcohol drinking, and a range of socioeconomic and demographic characteristics.

Our study is the first cross-sectional, population-based study to look at frequency of fish consumption and prevalence of diabetes in an Indian adult population and adds to the limited data on the associations between frequency of fish intake and diabetes prevalence in developing countries. However, data from previous studies of the relation of fish intake to diabetes risk are inconclusive. Accumulated evidence generated from a recent meta-analysis [29] of data from 438,000 individuals in 12 independent prospective cohorts with an average 11-year follow-up does not support an overall inverse association of fish or fish oil intake with the incidence of diabetes. An inverse association between fish intake and diabetes incidence was also found by combining studies conducted in Eastern (such as China and Japan) but not in Western countries (such as Finland, The Netherlands, the UK, and the United States) [29]. Another recent systematic review and meta-analysis [30] that included 527,441 participants and 24,082 diabetes cases reported heterogeneity between geographical regions in observed associations of fish consumption and risk of type 2 diabetes. The study found, for each serving per week increment in fish consumption, the relative risks (95% CIs) of type 2 diabetes were 1.05 (CI, 1.02–1.09), 1.03 (CI, 0.96–1.11), and 0.98 (CI, 0.97–1.00) combining US, European, and Asian/Australian studies, respectively [30].

Inconsistencies in the observed effect of fish consumption and diabetes in different populations including India may also reflect different preparation methods. In India, fish is either eaten dried, fried, or fried and then cooked with vegetables, gravy, or lots of spices, condiments, and cooking oil. It may be that the method of fish preparation (frying) and the type and amount of cooking fat used and the accompanying condiments with which fish is often served in India may not be beneficial for diabetes rather than the fish itself. Frying fish, especially deep frying, might produce transfatty acids, which might modify the beneficial effect of fish. Fried fish was not significantly associated with diabetes risk in a UK study [9]. In an earlier report from the same study [9], oily fish intake was associated with lower glycated hemoglobin, whereas another study [13] reported fried fish to be associated with higher glycated hemoglobin. In addition, the effect of fish intake on glucose metabolism may differ according to cooking method.

Studies reported that compared to raw fish, deep-fried fish intake is associated with higher concentrations of contaminants [31] and may reduce the potential for favorable health effects due to a reduction in eicosapentaenoic and docosahexaenoic acids [32]. Patel et al. [9] observed an inverse association of type 2 diabetes with non-fried (fresh, frozen, or canned) fish intake but not with fried fish. The high consumption of non-fried fish in Japan might partly account for the inverse association between fish intake and type 2 diabetes in a recent study [33]. Salting and drying, which are used to preserve fish, can also modify the association between fish intake and prevalence of diabetes. A salty diet could deteriorate insulin metabolism [34], and the drying of fish may accelerate the oxidation of polyunsaturated fatty acids, which in turn induces inflammation [35] and is a known predictor of type 2 diabetes [36]. Greater shellfish intake has been found to be associated with increased risk of diabetes in some studies [9] and the coastal states of India where plentiful sea/saltwater/shellfish are available are also the states where diabetes prevalence is higher [17] (see Appendix, Table A4).

Environmental contaminants including low-level exposure to some persistent organic pollutants has recently become a focus because of their possible link with the risk of diabetes [37]. Studies reported that some environmental contaminants found in fish have been associated with higher diabetes risk in US populations [38,39] but Villegas et al. [12] analyzed saltwater fish and freshwater fish separately to account for possible contamination of river water and found no evidence of a detrimental effect of freshwater fish consumption on the risk of diabetes. The contamination of freshwaters with a wide range of pollutants has become a matter of concern over the last few decades in India [40–42]. The natural aquatic systems in India have been extensively contaminated with heavy metals released from domestic, industrial, and other manmade activities [43]. Heavy metal contamination has devastating effects on the ecological balance of the recipient environment and a diversity of aquatic organisms [45,46]. Studies from India show that there has been a high accumulation of heavy metals in freshwater fishes [41,46–52], which might explain the positive association between fish intake and diabetes in our study. For example, investigations [46] on the accumulation of heavy metals (Cu, Ni, Fe, Co, Mn, Cr, and Zn) were carried out on 3 commercially important fish, namely, murrel, catfish, and carp, in a north India market revealed that the Fe and Zn concentrations were the highest in all tissues analyzed, followed by Ni, Cu, Co, Mn, and Cr in almost all 3 species. Another study [52] evaluated the annual variation in Zn, Mn, Cu, Pb, and Cd concentrations found in muscle tissue of 4 fish species (*Lates calcarifer*, *Mugil cephalus*, *Arius thalasinuss*, *Tilapia mossambica*) of the polluted Uppanar River at Cuddalore in Tamil Nadu (India) in relation to that of river water sampled during the dry season summer (March–June) of 2010 and 2011. The results revealed that the average concentrations of the trace metals in the fish were in following order: Zn > Mn Cu > Pb > Cd [52]. The concentrations of the metals in the water and muscle tissue of fish at downstream were many times higher than those in the upstream of the river. Almost all of the heavy metals (Cu, Pb, Cd, Zn, and Mn) concentrations in the river water exceeded the permissible limits of Indian standards and the Cu, Pb, and Cd concentrations in the muscle tissues of any of the fish species exceeded the provisional tolerable weekly intake [52]. The typical size of fish eaten in India is different in rural and urban India. In rural India, people mostly prefer small fish, but the urban Indian population prefers larger fish. Larger fish would have more toxic substances in their body than smaller ones due to bioaccumulation, which might partly explain the high prevalence of diabetes in urban Indian population. Because diabetes prevalence is rapidly increasing in India and the urban population, further study of the possibility that exposure to persistent organic pollutants contributes to the etiology of diabetes is critical.

Fish is an assumed source of beneficial dietary protein, and recent studies showed that dietary protein intake, despite its known beneficial effects on weight loss, may in fact increase insulin resistance and the risk of developing type 2 diabetes [53], which might be true for the positive association found in our study. Many people, and particularly patients with diabetes, will not sustain initially achieved weight loss, regardless ofthe chosen dietary strategy [54,55]. Our study showed that a causal association was unlikely, but there is increasing evidence that dietary protein intake, under isoenergetic conditions, may indeed increase insulin resistance via activation of the mTOR/S6K1 signaling pathway [56,57].

We separately conducted a cross-spousal sensitivity analysis of fish consumption and diabetes status. If fish eating is a proxy for a social exposure (e.g., wealth), we would predict that those not eating fish would have the lowest prevalence of diabetes and those who consume fish would have the highest. The cross-spousal result suggests a noncausal interpretation of the main finding. If fish eating was causally associated with diabetes, the effect of a husband's fish consumption on wife's diabetes and wife's fish consumption on husband's diabetes would have null odds ratios, whereas the direct associations of a wife's consumption on wife's diabetes prevalence and husband's consumption on husband's diabetes would have increased odds ratios. The adjusted ORs are all attenuated in our analysis, which would be expected particularly if the crude associations are due to confounding. It is likely that residual and unmeasured confounding explains the findings and suggests that there is no strong evidence for a causal association between fish consumption and diabetes status in Indian populations.

### Strengths and Limitations of the Study

The strengths and limitations of this investigation also merit consideration. The strengths of our study include the use of large nationally representative study sample, which allows comparisons to be made between men and women and the ability to examine this association in an adult Indian population. In addition, rigorous precautions were taken in the NFHS to obtain reliable self-reported data: the survey used local terminology and commonly understood terms to describe the disease, rigorously trained interviewers and supervisors, and standard quality checks. The survey was conducted using an interviewer-administered standardized questionnaire in the native language of the respondent and a total of 18 languages were used in the survey with back-translation into English to ensure accuracy and comparability [21].

The study has some limitations. The misclassification of dietary information in NFHS-3 data, although unavoidable, would most likely not allow for true associations. In addition, there is a possibility that the information derived from the NFHS-3 questionnaire, though critical to measure true dietary intake, is self-reported (instead of the use standard food frequency questionnaire or use of 24-hour recall method where the respondents have to estimate typical intake frequencies of food items and their portion sizes) and thus may not meet the standards of validity [58] despite the fact that NFHS-3 is a part of the DHSs conducted in more than 90 countries since the 1980s. Daily fish intake is rather low in India (6.5% among the sample respondents), which limited the possibility of studying the effects of high fish intake on diabetes prevalence. A higher fish intake was also associated with higher socioeconomic status in India; therefore, the participants in our study with higher exposure would also have been more likely to have regular health checkups. Given the high proportion of undiagnosed diabetes in developing countries (see http:// www.worlddiabetesfoundation.org) where less than half of people with diabetes are diagnosed, there is a possibility that the exposure was associated with the likelihood of testing for diabetes, which may result in detection bias.

The prevalence of diabetes in this large nationally representative survey of adult people was comparatively low (about 1%), reflecting the young age of this population and the use of self-report rather than physician diagnosis or biochemical assessment [17]. We were also unable to distinguish between type 1 and 2 diabetes diagnoses because there was no clinical confirmation on the reported cases. In most urban parts of India the health system is well developed enough for diagnosis of symptomatic diabetes, but at younger ages (<30 years) diabetes may not be symptomatic and NFHS-3 prevalence estimates are undoubtedly conservative, particularly for rural India where diagnosis may be much less likely to occur. Using self-reported data may be a source of bias, especially in rural areas, due to factors such as lack of awareness, low educational status, limited access to health services, and hesitation in disclosing diagnosed diseases. However, this ascertainment bias is unlikely to have been differential with respect to fish consumption. In addition, previous research has shown good agreement for self-reported diabetes when compared with medical records in a US population [59] and that self-reported health conditions demonstrate the expected relationship with socioeconomic status in India [60]. In addition, our analyses considering respondents who reported an unknown diabetes status were nearly identical to the main analyses (data not shown). Although our sample was relatively young (<50 years for women and men both), it is representative of the young population of profile of India; 84% of the Indian adult population (18–69 years) and 47% of the total Indian population at all ages fall within the ages covered by this study [61]. Our study does exclude approximately 14% of the Indian population (men and women over the age of 50) due to the sample design of the NFHS. The prevalence of diabetes increases with age, and whether a similar socioeconomic and demographic characteristics–diabetes relationship exists among middle and older age groups in all parts India is not clear [62], although our findings are consistent with the previous studies that have included older age groups.

Because this is a cross-sectional study, the entire study was with known diabetic subjects who might have altered their diet due to dietary advice based on diabetes control and the complications of diabetes. Therefore, the dietary choices of self-reported subjects with diabetes might have been modified to manage diabetes. Valid data on physical activity were not available in the NFHS-3, which is a limitation of this study because persons with healthier diets may be more physically active than others [7], and the lack of physical activity data in particular may have confounded the results. It is, however, possible that physical activity has in part been accounted for indirectly by adjusting for body mass index. In the present study, adjustment for socioeconomic and demographic factors, residential location, religion, and caste/tribe status of the respondents did not markedly modify the adjusted result, suggesting that the associations found are not completely explained by nondietary lifestyle factors.

## CONCLUSIONS

In a large nationally representative sample of adult men and women in India, significant positive associations between daily and weekly fish intake and diabetes were observed. However, this is an observational finding and uncontrolled confounding cannot be excluded as an explanation for the association; thus, these findings need further validation by longitudinal and clinical studies but may well have public health significance for the Indian population. More epidemiological research with better measures of frequency of food intake and clinical measures of diabetes are needed to validate the findings in a developing country.

### Author Contributions

S.A. conceived the article. S.A. conducted and S.E. supervised the statistical analysis. S.A. wrote the article and C.M., S.V., and S.E. revised it for important intellectual content. All authors gave final approval. S.A. and S.E. are the guarantors of this work and, as such, had full access to all of the data in the study and take responsibility for the integrity of the data and the accuracy of the data analysis.

## ACKNOWLEDGMENTS

An earlier version of the article was presented as a poster at the IEA World Congress of Epidemiology, Edinburgh, Scotland, 7–11 August 2011.

## FUNDING

S.A. is supported by a Wellcome Trust Strategic Award Grant No. Z/041825. C.M. is funded by the Higher Education Funding Council for England and the National Institute for Health Research Collaboration for Leadership in Applied Health Research and Care scheme. The authors acknowledge the support of International Institute for Population Sciences and Macro International (http://www.measuredhs.com) for providing access to the 2005–2006 Indian National Family Health Survey data.

## APPENDIX

**Table NaN:** **Table A1** Fish Consumption Pattern among Men and Women, India, 2005–2006

Fish Consumption	Men, *N* (%)	Women, *N* (%)	Total, *N* (%)
Daily	3706 (6.5)	6505 (6.5)	10,211 (6.5)
Weekly	14,414 (25.4)	22,070 (22.2)	36,484 (23.3)
Occasionally	21,818 (38.5)	34,242 (34.4)	56,060 (35.9)
Never	16,782 (29.6)	36,724 (36.9)	53,506 (34.2)
Total	56,720	99,541	156,261

**Table NaN:** **Table A2** Fish Consumption Pattern According to Consumption of Specific Food Items, Body Mass Index, and Other Lifestyle Indicators and Background Characteristics among Men and Women Aged 20–49 Years, India, 2005–2006

	Fish Consumption							
	Men (*n* = 56,742)				Women (*n* = 99,574)			
Characteristics	Daily	Weekly	Occasionally	Never	Daily	Weekly	Occasionally	Never
Consumption of specific food items								
Milk or curd								
Daily	31.4	40.6	41.4	61.0	35.5	35.4	33.1	51.4
Weekly	23.1	20.0	21.9	18.1	15.9	14.5	15.2	15.4
Occasionally	35.2	30.1	29.1	16.5	34.2	35.5	39.9	25.0
Never	10.3	9.3	7.6	4.3	14.5	14.6	11.8	8.2
Green leafy vegetables								
Daily	63.8	66.9	56.4	57.5	55.5	72.7	63.3	62.0
Weekly	27.5	28.9	36.8	36.1	29.1	23.6	29.9	30.8
Never/occasionally	8.7	4.2	6.8	6.4	15.4	3.8	6.8	7.3
Fresh fruits								
Daily	25.5	15.6	8.6	13.3	20.9	15.0	8.0	14.7
Weekly	32.0	36.7	31.7	35.6	32.8	29.5	24.2	26.7
Occasionally	40.3	44.8	57.0	48.2	43.0	51.9	64.4	54.7
Never	2.1	2.8	2.6	2.9	3.2	3.6	3.4	3.9
Pulses and beans								
Daily	45.2	55.3	51.2	53.8	43.2	52.0	52.6	54.9
Weekly	37.8	34.5	39.5	40.0	39.7	37.2	35.8	36.9
Occasionally	15.2	8.9	8.6	5.5	15.2	9.5	10.8	7.8
Never	1.8	1.3	0.6	0.7	1.9	1.3	0.8	0.5
Chicken or meat								
Daily	8.7	1.2	0.8	0.3	5.6	0.8	0.7	0.1
Weekly	41.3	60.9	19.6	6.1	34.7	58.2	15.4	4.3
Occasionally	47.2	35.7	77.3	14.2	54.5	36.9	80.2	8.3
Never	2.9	2.3	2.4	79.4	5.1	4.1	3.6	87.3
Eggs								
Daily	22.0	8.0	3.2	1.6	17.4	5.9	2.2	0.7
Weekly	48.2	70.0	31.7	11.2	41.9	67.2	25.6	6.7
Occasionally	23.4	18.4	60.5	18.2	31.5	20.2	65.1	10.4
Never	6.4	3.7	4.6	68.9	9.1	6.6	7.1	82.1
Body mass index and lifestyle factors								
Body mass index (kg/m^2^)								
≤18.5 (Underweight)	16.8	20.9	24.7	21.4	19.2	26.2	30.0	23.8
18.5–22.9 (Normal)	52.5	56.5	57.6	55.1	44.2	47.5	50.7	49.5
23.0–24.9 (Overweight)	16.0	11.3	8.9	10.9	12.8	10.9	8.2	10.4
≥25.0 (Obese)	14.6	11.3	8.9	12.6	23.8	15.4	11.2	16.3
Current tobacco smoking								
No	52.6	61.2	60.8	67.7	99.2	98.5	97.5	98.4
Yes	47.4	38.8	39.2	32.3	0.8	1.5	2.5	1.6
Alcohol consumption								
Never	55.3	55.7	56.2	81.2	98.3	97.0	95.6	99.5
Occasionally	26.5	25.1	27.9	14.0	1.2	1.3	1.8	0.3
Once a week	11.9	14.2	11.8	3.6	0.3	1.3	1.9	0.2
Almost every day	6.3	5.0	4.1	1.2	0.2	0.5	0.7	0.1
Frequency of watching TV								
Not at all	10.6	13.9	21.6	20.2	21.9	28.7	43.0	35.2
Less than once a week	16.1	18.6	23.7	17.7	10.2	9.8	11.9	9.6
At least once a week	17.9	17.9	15.0	15.3	11.5	11.8	10.0	11.4
Almost every day	55.5	49.7	39.7	46.7	56.4	49.7	35.1	43.8
Background factors								
Age								
20–29	38.7	41.7	39.7	40.0	40.9	45.2	43.8	42.3
30–39	34.7	33.5	34.3	32.5	34.7	32.7	34.4	33.4
40–49	26.6	24.8	26.0	27.5	24.3	22.0	21.8	24.3
Education								
Illiterate	11.8	18.2	26.5	16.5	20.4	38.5	57.4	42.6
Literate, less than middle school	17.1	18.9	19.7	14.1	16.7	17.1	13.3	13.7
Middle school completed	52.5	48.8	42.9	50.3	49.4	36.2	24.7	32.7
High school complete and above	18.6	14.0	11.0	19.1	13.5	8.1	4.7	11.0
Religion								
Hindu	73.6	78.4	80.1	90.6	69.7	75.3	76.7	90.4
Muslim	18.8	15.3	14.8	4.2	21.9	18.3	18.2	3.4
Christian	6.7	3.6	2.2	0.3	7.8	4.3	2.8	0.3
Sikhs	0.4	0.8	1.5	3.2	0.0	0.1	0.7	4.2
Others	0.5	1.9	1.4	1.6	0.6	1.9	1.7	1.6
Caste/tribe								
Scheduled caste	20.4	20.2	21.7	13.5	16.4	20.5	21.2	14.7
Scheduled tribes	3.8	9.3	10.9	5.2	3.2	8.6	11.3	5.5
Other backward class	26.7	33.9	41.7	42.5	23.9	32.9	41.9	42.7
General	36.8	31.5	23.5	37.9	48.1	33.0	22.6	36.2
Missing caste	12.3	5.1	2.2	0.9	8.4	5.0	3.0	0.9
Wealth index								
Lowest	8.1	14.8	21.3	12.0	8.3	15.7	24.7	13.1
Second	13.4	17.2	20.3	16.7	13.3	18.5	21.6	16.8
Middle	21.0	20.6	21.2	18.7	17.6	20.8	20.5	18.9
Fourth	26.8	25.2	19.8	22.0	28.3	22.9	18.4	21.0
Highest	30.7	22.3	17.3	30.6	32.5	22.0	14.8	30.2
Place of residence								
Urban	42.1	40.8	32.6	37.1	38.6	37.3	29.7	33.9
Rural	57.9	59.2	67.4	62.9	61.4	62.7	70.3	66.1
Number of cases	3,707	14,414	21,818	16,782	6,504	22,069	34,242	36,725

**Table NaN:** **Table A3** Bivariate Association between Wife's and Husband's Fish Consumption Pattern in India

	Never, *N* (%)	Daily, *N* (%)	Weekly, *N* (%)	Occasionally, *N* (%)	Total
Fish Consumption	Wife's fish consumption				
Both consuming	0 (0.0)	2360 (28.3)	5986 (71.7)	0 (0.0)	8346
Only wife	0 (0.0)	362 (8.9)	3693 (91.1)	0 (0.0)	4055
Only husband	1076 (22.3)	0 (0.0)	0 (0.0)	3759 (77.7)	4835
Both not consuming	11,871 (54.0)	0 (0.0)	0 (0.0)	10,129 (46.0)	22,000
Total					39,236
	Husband's fish consumption				
Both consuming	0 (0.0)	2304 (27.6)	6042 (72.4.0)	0 (0.0)	8346
Only wife	459 (11.3)	0 (0.0)	0 (0.0)	3594 (88.7)	4053
Only husband	0 (0.0)	346 (7.1.0)	4494 (92.9)	0 (0.0)	4840
Both not consuming	9718 (44.2)	0 (0.0)	0 (0.0)	12,287 (55.8)	22,005
Total					39,244

**Table 4:** **Table A4** Sample Distribution, Fish Consumption Pattern, and Diabetes Prevalence in India and Its States, 2005–2006

		Fish Consumption			
States	Sample Distribution, *N* (%)	Never, *N* (%)	Daily/Weekly, *N* (%)	Occasionally, *N* (%)	Diabetes Prevalence, *N* (%)
Northern region					
Jammu and Kashmir	1501 (1.0)	601 (40.0)	66 (4.4)	834 (55.6)	8 (0.5)
Himachal Pradesh	941 (0.6)	658 (70.0)	28 (3.0)	254 (27.0)	9 (1.0)
Punjab	4106 (2.6)	2982 (72.6)	271 (6.6)	852 (20.8)	42 (1.0)
Uttaranchal	1219 (0.8)	535 (43.9)	94 (7.7)	589 (48.4)	14 (1.1)
Haryana	3048 (1.9)	2559 (84.0)	73 (2.4)	415 (13.6)	35 (1.1)
Delhi	2090 (1.3)	1102 (52.8)	222 (10.6)	764 (36.6)	38 (1.8)
Rajasthan	8148 (5.2)	6562 (80.5)	172 (2.1)	14,115 (17.4)	30 (0.4)
Central region					
Uttar Pradesh	22,462 (14.4)	10,784 (48.0)	1689 (7.5)	9988 (44.5)	117 (0.5)
Chhattisgarh	3344 (2.1)	711 (21.3)	720 (21.5)	1913 (57.2)	31 (0.9)
Madhya Pradesh	9414 (6.0)	5211 (55.4)	928 (9.9)	3275 (34.8)	60 (0.6)
Northeastern region					
Sikkim	96 (0.1)	17 (17.9)	22 (23.2)	56 (58.9)	2 (2.1)
Arunachal Pradesh	159 (0.1)	5 (3.1)	86 (54.1)	68 (42.8)	1 (0.6)
Nagaland	208 (0.1)	3 (1.4)	83 (39.9)	122 (58.7)	2 (1.0)
Manipur	342 (0.2)	5 (1.5)	223 (65.4)	113 (33.1)	4 (1.2)
Mizoram	139 (0.1)	6 (4.3)	24 (17.4)	108 (78.3)	1 (0.7)
Tripura	589 (0.4)	7 (1.2)	486 (82.7)	95 (16.2)	14 (2.4)
Meghalaya	389 (0.2)	9 (2.3)	239 (61.4)	141 (36.2)	4 (1.0)
Assam	4368 (2.8)	102 (2.3)	3344 (76.6)	921 (21.1)	24 (0.5)
Eastern region					
Bihar	10,467 (6.7)	2049 (19.6)	1947 (18.6)	6468 (61.8)	135 (1.3)
West Bengal	13,439 (8.6)	337 (2.5)	11,336 (84.4)	1766 (13.1)	288 (2.1)
Jharkhand	3856 (2.5)	381 (9.9)	909 (23.6)	2565 (66.5)	30 (0.8)
Orissa	5960 (3.8)	362 (6.1)	3189 (53.5)	2407 (40.4)	55 (0.9)
Western region					
Gujarat	8176 (5.2)	5687 (69.6)	856 (10.5)	1629 (19.9)	80 (1.0)
Maharashtra	15,901 (10.2)	5698 (35.9)	4815 (30.3)	5379 (33.8)	125 (0.8)
Goa	254 (0.2)	18 (7.1)	224 (88.2)	12 (4.7)	7 (2.8)
Southern region					
Andhra Pradesh	12,352 (7.9)	1975 (16.0)	4297 (34.8)	6065 (49.2)	181 (1.5)
Karnataka	9596 (6.1)	3530 (36.8)	2189 (22.8)	3866 (40.3)	87 (0.9)
Kerala	4463 (2.9)	183 (4.1)	3893 (87.3)	384 (8.6)	142 (3.2)
Tamil Nadu	9288 (5.9)	1426 (15.4)	4267 (45.9)	3594 (38.7)	205 (2.2)
Number of cases	156,316	102,754		1771 (1.1)	

CI = confidence interval.
